# A mass casualty incident of infectious diseases at the port of Hamburg: an analysis of organizational structures and emergency concepts

**DOI:** 10.1186/s12995-021-00324-0

**Published:** 2021-08-31

**Authors:** Angelina Klein, Julian Heuser, Esther Henning, Nadine Sprössel, Ann-Christin Kordsmeyer, Marcus Oldenburg, Natascha Mojtahedzadeh, Jan Heidrich, Kristina Carolin Militzer, Lukas Belz, Thomas von Münster, Volker Harth, Lena Ehlers, Jens de Boer, Scarlett Kleine-Kampmann, Matthias Boldt, Martin Dirksen-Fischer, Lyubomir Haralambiev, Denis Gümbel, Axel Ekkernkamp, M. Sinan Bakir

**Affiliations:** 1 Department of Trauma and Reconstructive Surgery and Rehabilitative Medicine, University Medicine Greifswald, Ferdinand-Sauerbruch-Straße, 17475 Greifswald, Germany; 2 Institute for Occupational and Maritime Medicine (ZfAM), University Medical Center Hamburg-Eppendorf (UKE), Hamburg, Germany; 3 Hamburg Port Health Center, Institute for Hygiene and Environment, Hamburg, Germany; 4 Department of Trauma Surgery and Orthopedics, BG Hospital Unfallkrankenhaus Berlin gGmbH, Berlin, Germany

**Keywords:** Civil protection, Mass casualty incident, Maritime medicine, Emergency management, Medical hazard prevention, Port, Infectious outbreak

## Abstract

**Background:**

The project “ARMIHN” (Adaptive Resiliency Management in Port) focuses on strengthening the capability to act in a mass casualty incident (MCI) due to an outbreak of infectious diseases (MCI-ID). In addition to the current threat from the COVID-19 pandemic and associated outbreaks on cruise ships, previous MCI-ID were especially caused by pathogens such as Influenza virus or Norovirus. The first step was, to get an overview of processes and resources using the example of the Port of Hamburg, and to show the associated interaction of involved parties. This will serve as a basis for developing an operational strategy and offers the opportunity to optimize current work processes.

**Methods:**

A selective literature research using specified key words was performed and existing MCI concepts were received from local authorities. Identified structures and processes were analyzed in a multiple step process and also brought together through discussions in workshops with involved organizations and other experts. Additionally, the distances between the nearest rescue stations and selected hospitals from the Port of Hamburg were analyzed.

**Results:**

The current available concepts are proven, but an adaptation to an MCI-ID shows opportunities for a further cross-organizational development. The organizational structure of an MCI-ID in the Port of Hamburg was described, including a large number of involved organizations (*n* = 18). There are 17 involved fire and rescue stations and the port can be reached from these locations within 6 to 35 min. Based on their specialist expertise, 14 of the 31 listed clinics were selected.

**Conclusion:**

The purpose of the study was to provide an analysis of the current situation and show how involved parties would cope an MCI. A description of processes and resources at the Port of Hamburg will be used when designing a management plan for responding to an MCI-ID.

## Background

The threat posed by the current COVID-19 pandemic and associated outbreaks on ships like the Diamond Princess in February 2020, shows the urgency to develop concepts for infectious diseases at points of entry like ports in order to retain the ability to act in the event of an infectious disease (ID) outbreak [1, 2]. The continuous growth of the cruise ship sector, gives reason to be concerned about further outbreaks on ships and also shows the need to implement emergency plans [3]. Outbreaks do not have to be caused by novel pathogens, such as the severe acute respiratory syndrome coronavirus 2 (SARS-CoV-2). A mass outbreak of already known pathogens such as Influenza or Norovirus can also quickly push the medical care on ship and the subsequent rescue chain to their limits, due to a simultaneous high number of sick people [4, 5]. An analysis of existing structures and basic conditions on ships and in the port is the first step in creating an operational strategy for mass casualty incidents due to an outbreak of infectious diseases (MCI-ID). It forms the foundation and offers the possibility to identify areas for improvement.

Recent security research has increasingly dealt with the topic of mass casualties of injuries [6–11]. However, the existing emergency plans are primarily designed and tested for onshore use [12–21]. A MCI at sea or within a port, has rarely been focused [22, 23]. The literature mostly describes studies on outbreaks of infectious diseases on ships, while the rescue procedures in port are hardly reported [1, 2, 4, 5].

Knowledge of the existing structures and basic conditions in a port, as well as the ascertainment of the existing medical resources are necessary in order to manage an MCI-ID on a ship. By presenting the actual situation, optimization potentials can be worked out, and adapted hazard prevention can be ensured.

A first aim of the project “ARMIHN” (Adaptive Resiliency Management in Port) is to record the basic conditions and structures due to an outbreak of infectious diseases, including recommendations, guidelines, laws, and existing emergency concepts. In addition, existing (medical) resources in the port areas, rescue services and hospitals will be shown.

## Methods

The Port of Hamburg, Germany, is set in a highly industrialized infrastructure directly adjacent to the urban area and will serve as an example for the analysis. From a selective literature research including PubMed and Google Scholar databases, information was collected using the following keywords: “Maritime medicine”, “Mass casualty incident”, “Mass casualty of infectious diseases”, “Hamburg Port Health Center”, “Maritime Declaration of health”, “International Health Regulations”, “Rescue service Hamburg” “Fire Department Hamburg”, “Emergency concepts”, and “Hospitals Hamburg”. In this way international, national, and local recommendations, guidelines, legal texts, manuals and public documents from local institutions from their websites were included.

Additionally, non-confidential unpublished instructions and existing concepts for an MCI from local authorities were obtained. These are presented in a general approach as confidential material, personal and vulnerable data were excluded from this analysis. The collected documents for the analysis of the organizational structure and existing documents are demonstrated in a separate table (Table 1). Information was also collected through discussions with members of the fire and rescue department and the Hamburg Port Health Center. Current processes were also discussed in a workshop with 1–3 respective representatives from involved parties, including the Hamburg Port Authority, the Telemedical Maritime Assistance Service, ship pilots, the waterway police, aid organizations, and the German Seamen’s Mission. With the addresses of the rescue stations, the distance and arrival time was calculated using Google Maps® [24]. The port of Hamburg extends over a relatively large area, so a general fixed point in the center of the port was selected, which is used by Google Maps® after entering “Port of Hamburg”. The distance and travel time from the port to the hospitals was calculated the same way. Tables were created, then sorted by distance. For visualization, rescue stations and hospitals were presented on maps [24–26].

**Table 1:** Collected documents for the analysis of the organizational structure and existing concepts of an MCI in Hamburg [18, 27–39]

Collected documents for situation analysis in Hamburg	Included	Excluded
**Framework: Guidelines, recommendations, laws**		
The International Health Regulations (IHR)	X	
*Additional form for transmitted diseases (IHR)*		*X*
Handbook for management of public health events on board ships	X	
Handbook for Inspection of Ships and Issuance of Ship Sanitation Certificates	X	
Law for the prevention and control of infectious diseases in humans (Infection Protection Act - IfSG), Germany	X	
*Reporting form for infectious diseases (IfSG)*		*X*
*Order of domestic quarantine (IfSG)*		*X*
Fifth announcement of the status of medical requirements in maritime shipping (status of medical knowledge)	X	
Ship Occupation Ordinance (SchBesV)	X	
**Available Concepts**		
Emergency concept to cope with an MCI (MANV) Hamburg fire brigade	X	X Personal and vulnerable data
Alerting and response plan in the event of a health emergency of international concern in Hamburg	X	X Personal and vulnerable data
Flow chart for decision-making in the event of health emergencies in the Port of Hamburg	X	X Personal and vulnerable data
**Reporting**		
Maritime Declaration of Health (MDH)	X	
National Single Window (NSW)	X	
**Resources (material, infrastructure)**		
Strategy paper 2010 of the fire brigade Hamburg	X	
Annual report 2017 of the fire brigade Hamburg	X	
Hamburg Hospital Plan 2020	X	

The ARMIHN project is funded by the Federal Ministry of Education and Research Germany (13 N14925) and started in March 2019. The study was registered with the German Clinical Trials Register (DRKS; DRKS00022327) and approved by the local ethics committee (BB 051/19). All institutions and local authorities gave their informed consent in collecting and publishing data.

## Results

**Fig. 1:**
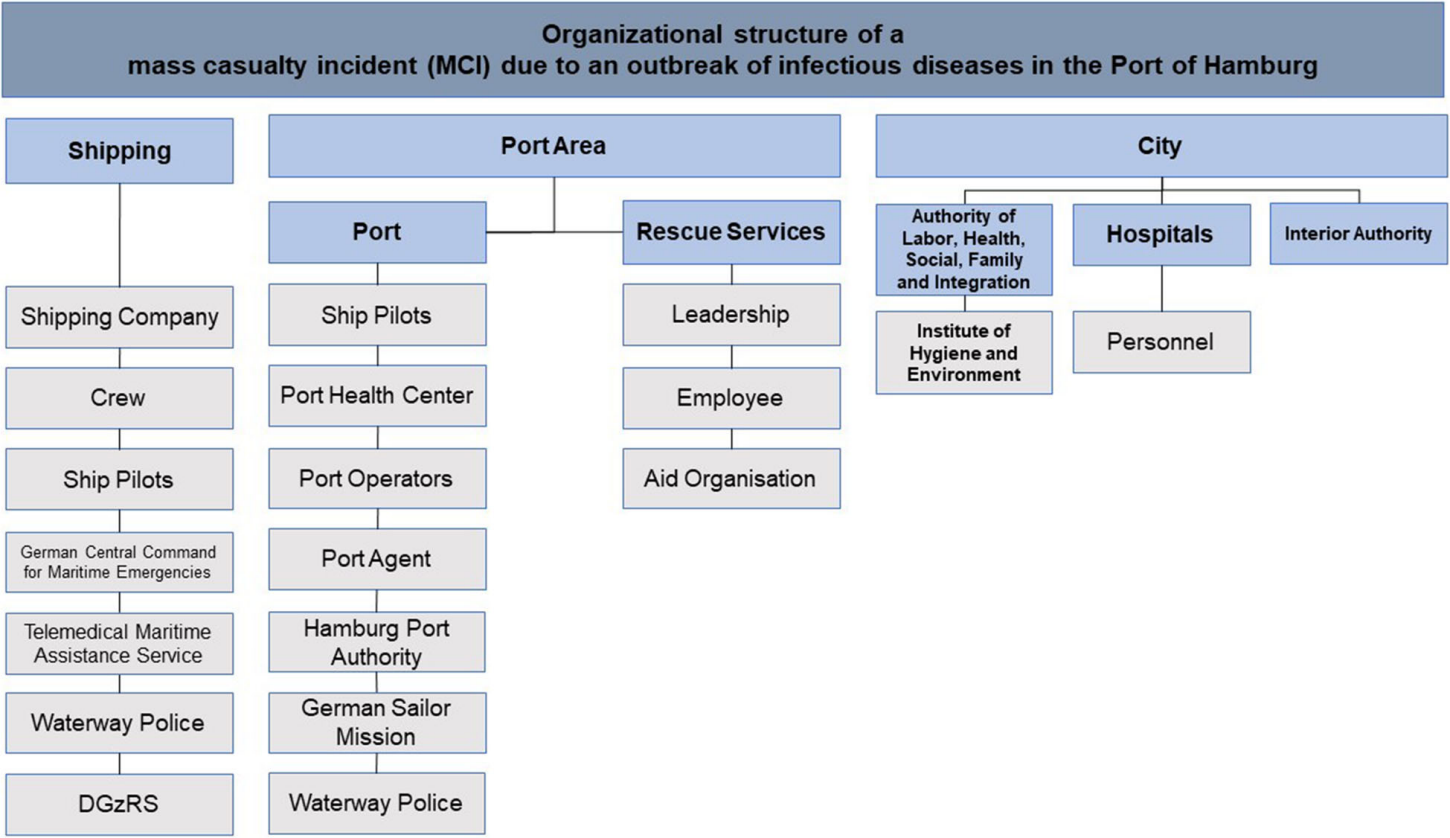
Organizational structure of a mass casualty incident (MCI) due to an outbreak of infectious diseases in the Port of Hamburg. DGzRS = Deutsche Gesellschaft zur Rettung Schiffbrüchiger (German Maritime Search and Rescue Association)

This study revealed that there are no documented experiences with MCI-ID in the Port of Hamburg until the beginning of the COVID-19 pandemic. In addition, the responsible authorities have not carried out any major damage exercises on this topic so far. During the project, an organizational chart of all these involved parties was developed, which reflects the complexity of MCI-ID at the port (Fig. 1). In general, the parties can be divided into different key areas. The section *Shipping* is represented by several organizations. The section *Port Area* is structured into two separated operational units, consisting of the *Port* and the *Rescue Services*. The section *City* is represented by several major players. The existing medical concepts (Hamburg Port Health Center, emergency service) are tested only by themselves within the organization and in some non-practical command-staff exercises [18, 27, 28].

### Guidelines

The diversity of the current state on maritime and health sectors include international, national and local laws, guidelines, and regulations for an infectious emergency on ships and in the port area. The International Health Regulations (IHR) published by the World Health Organization (WHO) ‘regulate the prevention of illnesses and form the basis of all further health regulations, also in response to the increase in international travel and trade’ [29]. A part of the implementation of the IHR in the maritime sector are manuals. They compromise topics relating to public health on ships, ship inspections and ship hygiene [30, 31]. They deal with hygiene standards, the implementation of WHO recommendations, and legal requirements on ships and ship inspection [4, 40].

In Germany, the Infection Protection Act regulates the investigation, protective measures, the observation, and the quarantine of infection events [32]. With these guidelines, an outbreak of an infection disease can be fought. Another basis concerning the resources of medical personnel is determined by the Federal Ministry of Transport and Digital Infrastructure, which constitutes the medical standard for ships flying under the German flag [33]. The Federal Office for Maritime Shipping and Hydrography also regulates whether a doctor and other medical personnel must be on board the ship [34]. Factors such as ship type, number of people on board, travel destination and duration are considered.

### Process of reporting infectious diseases in the port of Hamburg

For ships off and in German waters, the Telemedical Maritime Assistance Service or the German Central Command for Maritime Emergencies in Cuxhaven provides medical advices [41, 42]. This can be the first reporting station in the event of an outbreak if the ship is not located on inland water.

Regularly, a ship reports the current state of health of its persons on board via the Maritime Declaration of Health (MDH) at least 24 h before entering the port [35]. All cases of illness “since the start of the international trip or within the last 30 days” have to be listed in order to be able to draw conclusions about possible contamination and infection chains on board [35]. The National Single Window (NSW) is implemented as an electronic reporting system for ships entering a German port. Information such as the MDH can be made available to the competent authority via the system after a single report [36].

The Hamburg Port Health Center (HPHC) as the responsible authority in Hamburg will be informed about the MDH via the NSW. If there is a conspicuous MDH like a huge number of sick passengers or deaths, further information about the ship, the itinerary, and the measures already taken, can be obtained from the responsible port agent or the ship. If there is an MCI-ID, the HPHC can initiate various measures based on the initial assessment, inspection, and, if necessary, the expertise of the Hamburg Institute for Hygiene and the Environment. Crisis management documents, which regulate further alerting, the reaction, and decision making in the event of health emergencies in the port of Hamburg are available [27, 28]. A Hamburg-specific interdisciplinary committee called the “Fachstab Seuchenschutz” (Disease Control Unit) can be alerted in the event of possible epidemic or pandemic incidences [27, 28]. Experts from different specifications evaluate the emergency situation, advise and give recommendations for action and instructions. As one of five designated ports in Germany, the HPHC keeps protective clothing (smocks, gloves, mouth protection) and medical materials for sampling and diagnostics.

### MCI infrastructure of Hamburg

**Fig. 2:**
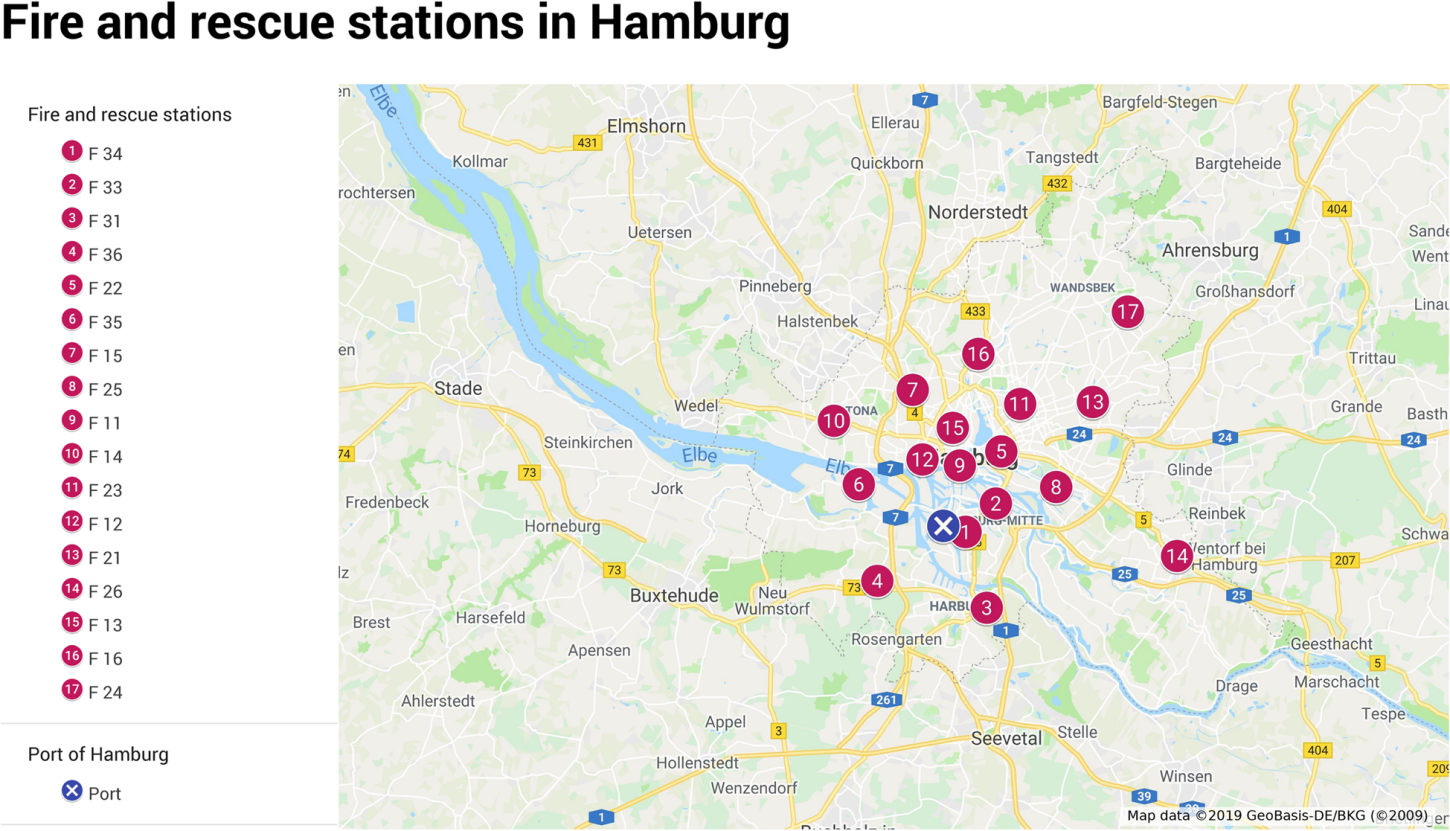
Fire and rescue stations in Hamburg [25]

If patients on a ship need to be hospitalized, the HPHC alerts the Hamburg Fire Brigade, which is responsible for providing emergency and rescue services in the city. The fire department is represented in Hamburg with 17 fire and rescue stations [37, 38]. The rescue workers reach the port from the different locations in an average of 6 to 35 min (Fig. 2).

The fire department has several available vehicles to deal with an MCI. They have four material-carrying equipment vehicles to build up treatment stations, two large-scale ambulances to transport easily ill patients and six vehicles called “GW- MANV” which carry material to treat up to 10 patients. All over the city, there are 124 ambulances. For high-infectious patients there are two special vehicles called “IRTW”. They have a sealed, easy-to-disinfect interior and special filter systems to prevent pathogens from getting into the environment [37, 38]. There are instructions on how to cope with an MCI, additionally to material resources [18]. It regulates the alert levels as well as the steps of the first arriving forces, the organization of deployment, types of alarm, the patient distribution and documentation, and the communication at the place of operation.

**Fig. 3:**
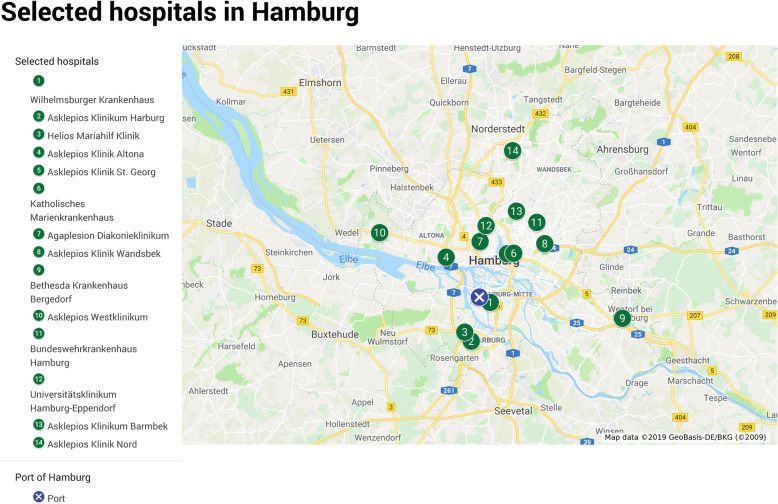
Selected hospitals in Hamburg with appropriate specialist departments for the treatment of infectious patients with an intensive care unit and a department for internal medicine [26]

The rescue control center and the head of operations on-site organize the distribution of the patients after examining the capacity of the hospitals. The Hamburg Hospital Plan 2020 lists 31 clinics in the city with a total inpatient bed capacity of 12,294 beds [39]. An exact number of currently existing and available isolation rooms or beds cannot be shown because the occupancy of the hospitals varies daily. Due to the focus of research in relation to the MCI-ID, clinics with appropriate specialist departments for the treatment of infectious patients have been selected (Fig. 3). Inclusion criteria for these clinics have been an intensive care unit and a department for internal medicine. Accordingly, purely surgical clinics are not included. Table 2 shows the total bed capacity of the selected hospitals as well as the average arrival time and distances from the Port of Hamburg to the hospitals [24, 26, 39].

**Table 2:** Selected hospitals and their total bed capacity, average arrival time, and distance to the Port of Hamburg [24, 26, 39]. The hospitals are sorted by average arrival time

Hospitals	Total bed capacity	Average arrival time (min)	Distance (km)
Wilhelmsburger Krankenhaus Groß-Sand	209	5	2,6
Asklepios Klinikum Harburg	918	13	9
Helios Mariahilf Klinik	176	14	11,3
Asklepios Klinik Altona	633	16	12,4
Asklepios Klinik St. Georg	695	19	10,3
Katholisches Marienkrankenhaus	586	20	10,7
Agaplesion Diakonieklinikum	388	24	19,5
Asklepios Klinik Wandsbek	553	25	15,1
Bethesda Krankenhaus Bergedorf	352	26	25,5
Asklepios Westklinikum	523	28	21,1
Bundeswehrkrankenhaus Hamburg	154	29	16,5
Universitätsklinikum Hamburg-Eppendorf	1476	30	21,4
Asklepios Klinikum Barmbek	670	30	16,1
Asklepios Klinik Nord	1268	35	31,7

Experts from the Bernhard Nocht Institute for Tropical Medicine (BNITM) and the University Medical Center Hamburg-Eppendorf (UKE), which focusses on infectiology, tropical medicine and gastroenterology, can treat patients in the treatment center for highly contagious infectious diseases (BZHI), as designated by the Robert Koch Institute (RKI). Highly contagious patients with infections and tropical diseases such as Ebola can be treated in special isolation rooms, which have their own locks [43, 44].

As a critical part of their infrastructure, hospitals are required to maintain their own alarm and response plans in preparation for major damage situations [10]. The Hamburg Hospital Act already wrote the emergency care in the hospital in April 1991 and requires the creation of alarm plans for external and internal large-scale operations [45]. The Authority of Labor, Health, Social, Family and Integration has been carrying out regular exercises in hospitals in order to be prepared for an MCI since 2005. This includes the construction of additional treatment areas (for example tents), triage and treatment of patients. These experiences and practiced processes can also be transferred to an MCI-ID with a necessary adaption concerning the different triage and treatment of the patients.

## Discussion

In this first step of the ARMIHN project, it could be shown that there are structures for coping an MCI-ID in the port, but that there is no universal, tried-and-tested concept. The relevance of the analysis is also shown by the fact that there is no experience of an MCI-ID in the Port of Hamburg, so far, and that this scenario has been rarely practiced in the port area yet. We were able to design an organizational chart that shows many involved parties and concurrently reflects the complexity.

It can be assumed that if more parties are involved in such a situation, there will be more challenges on the communicational and operational level [46]. Cooperation between different organizations in an emergency can only be efficient if existing concepts are brought together, coordinated, and most importantly tested regularly. In addition, the external conditions were listed by means of recommendations, laws and guidelines that have an influence on coping with an MCI-ID. The sub-chapters “Process of reporting infectious diseases in the Port of Hamburg” and “MCI Infrastructure of Hamburg” should give an impression of how an MCI is reported and which resources of the city of Hamburg are available, for example through hospitals and the fire brigade.

The special processes of the existing emergency concepts were not described in detail in order to protect important and vulnerable data from misuse. After extensive discussion with the residential committee for safety-relevant research in a separate meeting and as part of the approval of the local ethics committee we were only able to reproduce the relevant data in a general context in order to avoid a dual use risk. This represents a limitation of this work. Nevertheless, it was important to show that concepts exist and serve as the basis for developing a publishable, common concept in further project work.

European projects such as SHIPSAN ACT, SHIPSAN Trainet, and the Healthy Gateways Joint Action, provide initial approaches on the preparation and prevention, as well as the occupational health practices, of possible outbreaks of infectious diseases [4, 40, 47]. This year the EU Healthy Gateways working group published a guideline that is intended to support the development of public health concepts for ports and describe the procedure. According to recommendation, international and national laws and regulations should be known, existing operational plans should be integrated, communication should be regulated and the concept should then be tested and trained [48]. In this respect, the presentation of the organizational structures, existing concepts and resources is not only useful and necessary for current events of the COVID-19 pandemic, but also confirm the work of the ARMIHN project, which is currently developing such a concept for Hamburg. The Port of Hamburg can be approached in any emergency due to its special function as one of five designated ports in Germany and must therefore be prepared for it.

Our findings are not completely transferable to other national or even international ports, nevertheless, the basic patterns of the analyzed influencing factors, like the EU Healthy Gateways guideline shows, should be the same [48]. We assume that our project and this work can be an example for future transferring adapting strategies to similar facilities.

In addition to the new guideline, there are publications like an analysis of an operation on the high seas in 2012, which showed that the preparation, the determination and provision of resources, as well as training, are important factors for effectively coping with an MCI in the maritime setting [22]. Another study also shows that regular refreshments of seafarers’ medical skills are important in order to achieve a training effect and to be able to initiate appropriate treatment in emergencies [49]. A recent publication dealing with digital versus analogue record systems for MCI at sea underlines that improvements are also required there. The superiority of digital triage of patients over analogue was examined. It showed less mis-triage, which can be particularly dangerous for patients on the high seas [50].

It should be kept in mind that medical standards on ships are dependent on the country and the flag under which the ships sail. Therefore, there are discrepancies, and it might be not possible that same standards are being used overall. This affects both medical resources and staffing [33, 34, 40]. Regionally different MCI concepts must be taken into account, too [11–14, 17].

Thus, we recommend carrying out large-scale infection disaster exercises in the port that have not been implemented so far. Several organizations such as the HPHC, rescue services and all those involved ship and port workers are able to test and develop common processes in order to become reliable and resilient against an MCI-ID.

A cross-organizational task force that can be alerted in medical emergencies, such as the “Fachstab Seuchenschutz” (Disease Control Unit) in Hamburg, has various advantages [32, 40]. Through quick identification and assessment of the situation, decisions can be made quickly, which would serve to better deal with such a situation. In this way, the city of Hamburg aims at clarify competencies in advance and guarantee security of action in infectious emergencies.

The selection criteria made by an intensive care unit and internal specialist department is possible in a big city like Hamburg with at least 14 available clinics and their 8601 fully inpatient beds. In the event of resource shortages, it can be extended to surgical clinics to admit and treat patients [18, 25, 26, 37–39]. However, such a selection cannot be made everywhere, especially with regard to smaller municipalities and cities. Additionally, the distribution of patients could be optimized via a central (or regional) register of available beds for infectious patients, whereas in Germany IVENA (IVENA by mainis IT-Service GmbH, Offenbach am Main, Germany) is a potential system that is already used in a similar way and is the most widespread. By expanding this system, existing resources for COVID-19 patients can be displayed [51]. This can be linked and used analogously to the national register for intensive care capacities, which was implemented in the course of the COVID-19 pandemic and has gained in importance in Germany [52, 53].

The purpose of the ARMIHN project is to practice and to test the operational strategy through the modeling of damage scenarios and disease patterns, in three theoretical exercises and one practical exercise with all involved parties in future steps, so that a first training effect can be achieved. The developed operative strategy can be expanded in a future-oriented way to deal with a new type of pathogen such as SARS-CoV-2. The need for standardized cross-organizational concepts and the preparation for MCI-ID was underlined by the COVID-19 pandemic and associated outbreaks on ships.

## Conclusion

This research shows that coping an MCI-ID in the port is a multilayered complex collaboration involving several stakeholders from different organizations. Most organizations have their own concept for MCI without focusing infectious diseases, but there is still potential to uniform and practice the procedure. The external conditions such as the multitude of legal bases and laws, different medical standards and lacking cross-organizational concepts even complicate the situation. By identifying the existing structures, processes and resources, an emergency concept for an MCI-ID in the port can be improved and be tested for practical suitability. It can contribute to an improved resilience dealing with an MCI-ID.

## Data Availability

The datasets supporting the conclusions of this article are included within the article (and its additional files). Instructions and existing concepts for an MCI from local authorities (HPHC, Fire Brigade) are not publicly available, because of sensitive information and has been excluded from analysis.

## Abbreviations

ARMIHN: Adaptive Resiliency Management in Port (Adaptives Resilienz Management im Hafen)
BNITM: Bernhard Nocht Institute for Tropical Medicine
BZHI: Treatment center for highly contagious infectious diseases (Behandlungszentrum für hochansteckende Erkrankungen)
DGzRS: German Maritime Search and Rescue Association (Deutsche Gesellschaft zur Rettung Schiffbrüchiger)
HPHC: Hamburg Port Health Center
ID: Infectious disease
IHR: International Health Regulations
MCI: Mass casualty incident
MCI-ID: Mass casualty incident due to an outbreak of infectious diseases
MDH: Maritime Declaration of Health
NSW: National Single Window
RKI: Robert Koch Institute
SARS-CoV-2: Severe acute respiratory syndrome coronavirus 2
SHIPSAN ACT: EU Ship Sanitation Programme
SHIPSAN Trainet: EU Ship Sanitation Programme to develop training programs
UKE: University Medical Center Hamburg-Eppendorf (Universitätsklinikum Hamburg-Eppendorf)
WHO: World Health Organization
